# 

**Published:** 1892-03-12

**Authors:** 


[illegible], Vol. XI.—March 12th, 1892.

[illegible]red at the General Post Office as a Newspaper for [illegible]nission in the United Kingdom and Abroad.]

WITH EXTRA NURSING SUPPLEMENT.
Per Annum, 6s. 6d., post free.
Monthly Parts, 6d.; post free, 8d.
Ditto per Annum, 8s., post free.

### THE HOSPITAL

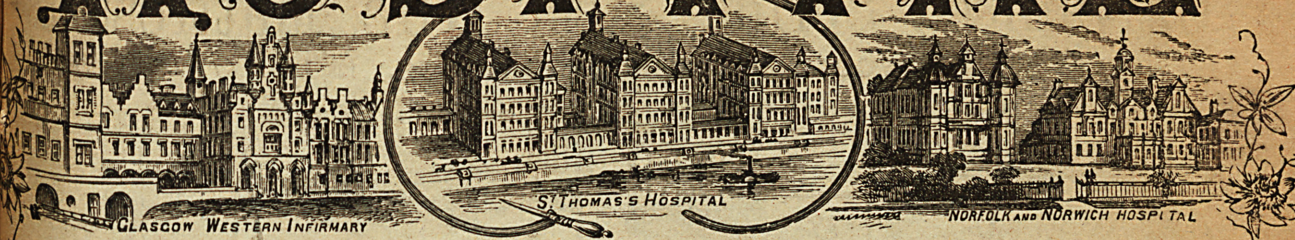

WITH EXTRA NURSING SUPPLEMENT.

No. 285. Vol. XI.] SATURDAY, MARCH 12th, 1892. [ONE PENNY.

